# Justice beliefs for self and others: Associations with positive and negative affectivity in African Americans and White Americans

**DOI:** 10.1371/journal.pone.0297762

**Published:** 2024-02-26

**Authors:** Todd Lucas, Isaac M. Lipkus, Ludmila Zhdanova

**Affiliations:** 1 Charles Stewart Mott Department of Public Health, College of Human Medicine, Michigan State University, Flint, Michigan, United States of America; 2 School of Nursing, Duke University, Durham, North Carolina, United States of America; 3 Department of Psychology, Adler University, Vancouver, British Columbia, Canada; St John’s University, UNITED STATES

## Abstract

Prior research has shown that a belief in personal justice (i.e., justice for self) is associated with better health and well-being, whereas a belief in justice more generally (i.e., justice for others) is unrelated. However, an emerging perspective is that racial differences may overlay the relationships between multidimensional beliefs about justice and indices of well-being. This includes that well-being among African Americans may be additionally supported by rejecting rather than endorsing some forms of believing in justice. In the present study, we consider racial similarities and differences in the links between beliefs about justice for self and others and emotional well-being. African Americans (*N* = 117) and White Americans (*N* = 188) completed measures of beliefs about justice for self and others, and also measures of dispositional tendencies towards experiencing positive and negative emotion (i.e., positive and negative affectivity). In both groups, beliefs about justice for the self were associated with greater positive affect and reduced negative affect. However, beliefs about justice for others were additionally associated with greater negative affect only among African Americans. The link between justice for others and negative affect among African Americans was not attributable to measurement or mean differences in justice beliefs across racial groups, or to socioeconomic differences. Results align with an emerging perspective that simultaneously endorsing and rejecting justice beliefs may be vital to preserving well-being for some racial minorities.

## Introduction

According to Lerner [1] people strive to believe that the world is a fair and just place. This *belief in a just world* can provide individuals with the psychological resources to live an orderly and structured life. Inspired by Lerner’s just world hypothesis, a vast literature has evolved to support that just word beliefs not only predict important social attitudes and behaviors, but also that a belief in justice can help individuals cope with the world in ways that protect and enhance personal well-being [2,3]. However, more recent research has illustrated that salutogenic links between justice beliefs and well-being are nuanced. Namely, whereas a belief that the world is fair and just to one’s own self is strongly associated with personal well-being, a belief that the world is fair generally or to others confers little or no personal health benefit [4–7].

Although a multidimensional perspective of justice illuminates specific links between believing in justice and well-being, an emerging perspective is that these relationships might also differ according to race or cultural factors. This includes among African Americans, for whom rejecting rather than endorsing some forms of justice also may play a role in maintaining well-being [8,9]. Possible racial differences not only encompass whether links between self justice beliefs and personal well-being are robust, but also whether beliefs about justice for others and well-being might be additionally associated among African Americans. In the present study, we compare links between justice beliefs for self and others and emotional well-being among African Americans and White Americans. In doing so, we provide evidence that simultaneously endorsing and rejecting beliefs about justice may be important to maintaining well-being among racial minorities, whose lived experiences may promote psychological adaptations to cope with social injustice.

### Justice beliefs, health and well-being

According to just world theory, people are universally driven by a preconscious need to believe the world is fair. However, belief strength also varies as a result of socialization and life experience, resulting in individual differences. Justice beliefs encompass the enduring tendencies of individuals to believe in fairness [10]. Although associations between justice beliefs and health can be complex e.g., [9,11], available literature has shown that believing in justice generally and usually carries a capacity to promote better health and well-being [2,3,12]. Indeed, justice research highlights the ways in which justice beliefs may protect physical health, including links to lower incidence of cardiovascular disease [13,14] better sleep [15] and also lower risk of metabolic disease [16].

Links from justice beliefs to mental well-being are also abundantly evident [3]. Of current interest, these links encompass indices of emotional well-being, and justice beliefs have been broadly shown to both promote positive affect and reduce negative affect [17–19]. Specifically, a strong belief in justice has been linked not only to greater life satisfaction and general positive mood [6,20–23], but also to reduced anxiety, depression, and psychological distress [7,24–26]. Importantly, links between justice beliefs and both positive and negative emotions are causal [19], and remain even after accounting for individual differences and beliefs that are conceptually related to justice [7]. From a mechanistic standpoint, effects of justice beliefs on emotional well-being are thought occur by enabling better stress coping [3,27]. In support, justice beliefs have been linked to biological indices of stress [28–31]. More recently, justice beliefs have also been linked to telomere length [9]–a sign of healthy aging that corrborates links between justice beliefs and better stress coping [32].

### Justice beliefs for self and others

Overlaying the capacity of justice beliefs to enhance well-being, theory and research also suggest that beliefs about justice operate in multiple spheres [1,6]. This includes beliefs about fairness accorded to others (i.e., general justice for others), as well as feelings about whether one personally gets what one deserves (i.e., personal justice for self). Research reveals not only that beliefs about justice for the self and others are psychometrically distinct [6], but also that these beliefs tend to be only moderately correlated with one another [33], and are cross-culturally observable [34,35]. Moreover, justice research supports that distinguishing between beliefs about justice for self and others is empirically useful [4–7,33,36]. Namely, beliefs about justice for self and others tend to be differentially correlated with health and social attitude measures–whereas beliefs about justice for others best predict measures of harsh social attitudes, beliefs about justice for the self better predict well-being [5,6].

### Justice beliefs and African American well-being

Although beliefs about justice for the self have been shown to link to personal well-being more strongly than beliefs about justice for others, little research to date has considered racial or cultural similarities and differences. This dearth includes African Americans, for whom thoughts about injustice may be an easily accessible and important determinant of well-being e.g., [8,37]. Moreover, and although justice perceptions among African Americans have been linked to health-affecting social treatment such as affirmative action [38,39], racial profiling [40], and interpersonal discrimination [41], direct links to well-being have been relatively overlooked.

Presently, we consider that in addition to protective health effects of believing in justice, rejecting some forms of this belief might confer salutogenic benefits for marginalized individuals. That is, well-being among African Americans may be best supported by simultaneously endorsing and rejecting particular beliefs about justice. Theoretical support for positive and negative associations between justice and well-being can be gleaned from inconsistency theoretical frameworks, which highlight that well-being is determined by the extent to which individual-level social expectations are concordant with actual social experience [11,42]. With respect to justice, inconsistency frameworks highlight that harmony between one’s justice beliefs and justice-related experiences, rather than a rote endorsement of justice, may fundamentally govern emotional health. Overlaying inconsistency theoretical frameworks, individual differences research highlights that for African Americans, justice beliefs might be both positively and negatively associated with health and wellness through differential links with justice beliefs for self and others. On one hand, believing the world is fair to oneself provides a universal coping resource by reassuring individuals that the world is orderly and predictable [1]. This belief in personal justice may be similarly helpful to racial minorities, who may also benefit from the sense of control that seeing the world as fair to oneself can provide [43]. However, marginalized individuals might additionally benefit from not endorsing a more general belief that the world is fair for others, especially to the extent that rejecting this belief may more accurately reflect attunement to social marginalization. Indeed, stress coping cultural frameworks have emphasized the notion of acknowledging injustice more generally, while also maintaining and acting on the belief in personal justice. For example, John Henryism [44] is a culturally enshrined coping strategy among African Americans that urges grit and personal accountability (i.e., a belief in justice for self) in an effort to overcome unfair life obstacles (i.e., belief in general injustice).

Empirical support for differentially linking beliefs about justice for self and others to emotional wellness is provided by studies that show racial minorities more readily acknowledge social mistreatment for others than for self [45], suggesting that African Americans may aim to deflect personal instances of injustice, but not general instances. Moreover, recent research on telomere length and cancer screening behavior both support that among older African Americans, stress coping afforded by the belief in personal justice may be most robust when accompanied by a weaker belief in justice for others [9,43] Finally, recent research suggests that sexual and racial minority men may experience lower perceived discrimination to the extent that personal justice beliefs more strongly exceed the belief in justice for others [46].

### The present study

With an eye towards exploring potential racial similarities and differences, the present study sought to compare links between justice beliefs and emotional well-being. Samples of African Americans and White Americans completed measures of beliefs about justice for self and others, and also trait measures of positive and negative affect. Using structural equation modeling and model comparison strategies, we examined whether links between justice beliefs for self and others and emotional well-being were cross-racially equivalent. Based on prior literature that has established a general and salutogenic effect on personal health, we expected beliefs about justice for self to be associated with increased positive affect and reduced negative affect in both racial groups (i.e., equivalent effects). However, and with an eye towards inconsistency framework literature and recent empirical work, we expected beliefs about justice for others to also be exclusively linked to greater negative affect among African Americans (i.e., non-equivalent effects).

## Method

### Participants and measures

This study included samples of African Americans (*N* = 117) and White Americans (*N* = 188). Participants were recruited via the internet and were entered into a lottery to receive a small retail prize as compensation. Table 1 summarizes demographic and socioeconomic information collected in both samples. All participants completed an online survey entitled “Perceptions of Daily Living” that required approximately ten minutes. We administered measures of dispositional justice beliefs and trait affect in both samples. Table 2 presents means, standard deviations, internal consistency coefficients, and bivariate intercorrelations.

**Table 1:** Sample demographic characteristics.

	African American (N = 117)		White (N = 188)	
	n	%	n	%
Gender				
Male	33	28.20	72	38.30
Female	83	70.90	116	61.70
Missing	1	.90	0	.00
Income (in thousands)				
0–19	17	14.53	23	12.23
20–39	42	35.90	46	24.47
40–59	26	22.22	52	27.66
60–79	16	13.68	26	13.83
80–99	10	8.55	14	7.45
100 up	6	5.13	27	14.36
Education				
High School	18	15.38	36	19.15
Some College	43	36.75	66	35.11
College Graduate	47	40.17	58	30.85
Advanced Graduate	8	6.84	27	14.36
Missing	1	.85	1	.53

Note. Annual household income reported in thousands.

**Table 2:** Means, standard deviations, reliability coefficients, and bivariate associations.

	Mean	SD	1.	2.	3.	4.	5.	6.	7.	8.	9.	10.
African American (*N* = 117)												
1. DJW-self	17.56	5.58	**.88**									
2. PJW-self	17.48	5.30	.65***	**.92**								
3. DJW-others	17.78	5.14	.42***	.48***	**.83**							
4. PJW-others	16.04	4.96	.54***	.68***	.63***	**.91**						
5. BJW-self	35.04	9.88	.91***	.90***	.49***	.67***	**.92**					
6. BJW-others	33.82	9.11	.53***	.64***	.91***	.90***	.64***	**.90**				
7. Positive Affectivity	38.36	7.27	.28**	.21*	.17	.16	.27**	.19*	**.92**			
8. Negative Affectivity	20.09	7.26	-.08	.00	.14	.08	-.05	.13	-.26**	**.83**		
9. Income	2.81	1.36	-.11	.00	-.24*	-.05	-.06	-.16	-.13	-.07	--	
10. Education	3.39	.83	-.15	-.21*	-.14	-.16	-.20*	-.17	-.07	-.10	.35**	--
White (*N* = 188)												
1. DJW-self	20.08	4.78	**.90**									
2. PJW-self	19.02	5.12	.64***	**.94**								
3. DJW-others	17.73	4.76	.49***	.43***	**.87**							
4. PJW-others	16.72	4.96	.43***	.65***	.61***	**.91**						
5. BJW-self	39.12	8.97	.90***	.91***	.51***	.60***	**.93**					
6. BJW-others	34.45	8.70	.51***	.61***	.89***	.90***	.62***	**.90**				
7. Positive Affectivity	34.91	7.12	.09	.18**	.10	.17*	.15*	.15*	**.90**			
8. Negative Affectivity	20.62	7.45	-.28***	-.36***	-.13	-.23**	-.36***	-.21**	-.23**	**.90**		
9. Income	3.23	1.56	.11	.11	.10	.10	.12	.11	.07	-.10	--	
10. Education	3.40	.98	.13	.03	.00	-.06	.08	-.04	-.05	-.08	.27**	--

Notes. BJW-others and BJW-self are higher order variables. Cronbach’s Alpha reported on diagonal in bold. Income and education are single item measures and thus do not report internal consistency values.

*p < .05

**p < .01

***p < .001.

### Justice beliefs for self and others

Justice beliefs were measured using a modified version of the Procedural and Distributive Just World Beliefs scale [47]. In its original form, this measure captures individual tendencies to see rules and treatment (procedural just world beliefs) and also outcomes and allocations (distributive just world beliefs) as deserved. Following the lead of others e.g., [6], beliefs about justice for self and others were measured by expanding the original eight-item measure to include 16 items [36]. Thus, justice beliefs for self and others were each indicated by two subscales. Procedural just world beliefs for self (PJW-self) measured beliefs about the deservedness of rules, processes, and treatment towards oneself (e.g., ‘I am generally subjected to processes that are fair’), whereas distributive just world beliefs for self (DJW-self) measured beliefs about the deservedness of outcomes or allocations for the self (e.g., ‘I usually receive outcomes that I deserve’). Similarly, procedural just world beliefs for others subscale (PJW-others) measured beliefs about the deservedness of rules and treatment for others (e.g., ‘Other people are generally subjected to processes that are fair’), whereas distributive just world beliefs for others (DJW-others) subscale measured beliefs about others’ outcomes or allocations (e.g., ‘Other people usually receive outcomes that they deserve’). All items were rated using a Likert-type scale ranging from 1 (*strongly disagree*) to 7 (*strongly agree*), with higher scores indicating a stronger belief in justice. Four subscale totals were created by summing appropriate items, and beliefs about justice for self and others were specified as higher order constructs that were each indicated by two subscales [36].

### Positive and negative affectivity

Dispositional tendencies towards experiencing positive affect (PA) and negative affect (NA) were measured using the Positive and Negative Affect Scale PANAS [48]. The PANAS consists of ten positive affect and ten negative affect adjectives. Participants rate the extent to which they experience each of twenty specific feelings and emotions. All items are rated using a Likert-type scale that ranges from 1 (*very slightly or not at all*) to 5 (*extremely*). Separate scores are calculated for positive and negative affect by summing together appropriate items. In the present study, participants provided trait measures of positive and negative affect (i.e., affectivity) by indicating the extent to which they generally experience each feeling or emotion.

### Overview of statistical analysis

Latent level structural equation modeling was used to examine relationships between justice beliefs and affectivity. Analyses were performed using LISREL 8.80 [49] and maximum likelihood estimation. In all instances, the covariance matrix was analyzed, and scale was set using the disturbance term of each latent variable. Acceptable fit was indicated by a non-significant chi-square goodness of fit test, Nonnormed Fit Index NNFI; [50] and Comparative Fit Index CFI; [51,52] values above .90, and Root Mean Square Error of Approximation RMSEA; [53] and Standardized Root Mean Square Residual SRMR; [54,55] values at or below .08 [56]. As is common with latent level structural equation modeling, we expected chi square indices to be significant. Therefore, we gave greater consideration to other fit indices and to model comparison strategies [57]. We conducted three sets of analyses. First, to rule out psychometric differences as a possible source of subsequently evaluated racial differences, we examined the measurement equivalency (i.e., invariance) of beliefs about justice for self and others across racial groups. Second, we specified and formally compared a series of structural models that linked beliefs about justice to measures of PA and NA among African Americans and Whites. Finally, to rule out socioeconomic variables as a viable explanation for any observed differences, we reanalyzed a final structural model while also controlling for education and income in both racial groups.

## Results

### Measurement equivalency of justice beliefs

We initially evaluated the psychometric equivalency of self and other justice beliefs using invariance testing e.g., [58–60]. Specifically, we tested the invariance of a four-factor justice model that specified separate but correlated latent variables for DJW-self, PJW-self, DJW-others, and PJW-others, with each of the four subscales indicated by the appropriate four subscale items [35,36]. Consistent with recommended practices, we specified separate covariance matrices and justice beliefs measurement models for African Americans and Whites [35]. We then examined changes in model fit produced by imposing increasingly stringent equivalency requirements on parallel items. Equivalency of justice beliefs across ethnicity was supported to the extent that increasingly constrained models fit as well as prior specified and lesser constrained models. Because chi square difference tests are overly sensitive to models with large degrees of freedom e.g., [61], we followed recommendations and compared models using changes in CFI. A worse fitting (i.e., non-invariant) model was formally indicated by a decrease in the CFI index of greater than .01 [59]. We began by assessing the configural equivalency of items—the least restrictive form of invariance. Configural invariance exists when parallel items load significantly onto the same constructs across groups. Configural equivalency provided a baseline for assessing increasingly stringent forms of invariance that included metric invariance (factor scores for each item are equivalent across groups), intercept invariance (equivalent item intercept values across groups), and mean invariance (equivalent scale means across groups). The equivalency of *structure* is comprised of configural, metric, and intercept invariance, while the equivalency of *level* is defined as scale mean invariance [62].

Table 3 presents fit indices and model comparisons. All models specified a four-factor structure with PJW-self, DJW-self, PJW-others, and DJW-others modeled as correlated latent factors [36]. The baseline configural model fit well, with the exception that the RMSEA was modestly below the conventional benchmark value for acceptable fit. All item loadings were positive and significant at *p* < .001. In the African American subsample, item loadings ranged from 1.15 to 1.31 for self justice beliefs, and from 1.03 to 1.31 for beliefs about justice for others. In the White subsample, item loadings ranged from 1.02 to 1.29 for self justice beliefs, and from .67 to 1.56 for beliefs about justice for others. Latent factor correlations ranged from .43 to .68 for the White subsample and from .50 to .73 for the African American subsample. Most importantly, model comparison strongly supported the psychometric equivalency of justice beliefs across ethnicity. Models that imposed increasingly stringent metric, intercept, and mean level invariance fit as well as prior specified models according to CFI change criteria. We thus concluded that beliefs about justice for self and others were psychometrically equivalent across racial groups in both structure and level.

**Table 3:** Invariance of justice beliefs in African American and white samples.

Model	χ^2^	*df*	NNFI	CFI	RMSEA	SRMR
						
1. Configural	735.18	196	.941	.952	.126	.070
2. Metric	742.77	212	.947	.953	.120	.082
3. Intercept	764.13	224	.949	.952	.118	.081
4. Mean	786.91	228	.948	.951	.119	.085
Model Comparisons		ΔCFI				
1 vs. 2		.001				
2 vs. 3		(.001)				
3 vs. 4		(.001)				

Notes. Value in parentheses indicates a decrease in ΔCFI. *p < .05, ***p < .001.

### Justice beliefs and affectivity

Links between justice beliefs and affectivity were examined by separately specifying and formally comparing multigroup structural models for African Americans and Whites, in which separate structural models for African Americans and Whites were simultaneously estimated as a single multigroup model. Two latent variables for beliefs about justice were specified [36]. Self justice beliefs were indicated by PJW-self and DJW-self subscale totals, and justice beliefs for others were indicated by PJW-others and DJW-others subscale totals. To preserve an acceptable ratio of observations per model parameter [63], variable parceling was utilized for PA and NA. Latent variables for each affectivity measure were indicated by two five-item parcels that were created using even and odd numbered subscale items. Prior to creating parcels, we verified a unidimensional factor structure for both PA and NA items by means of a principal components factor analysis [64]. Invariance testing again formed the basis for model comparison. To assess path invariance, we examined changes in model fit produced by incrementally constraining each of the four structural paths originating from self and other justice beliefs to PA and NA. Once identified, an invariant path was retained as such in all subsequently tested models. Similarly, a path that was identified as nonequivalent across ethnicity was allowed to vary in subsequent models. Chi square difference tests were used to examine changes in model fit. In all structural models, we allowed latent variable correlations between justice beliefs for self and others, and also between positive and negative affectivity.

Table 4 presents structural model comparisons, which were conducted using the single set of fit indices obtained for each multigroup model. The initial measurement model (model 1) fit well according to all indices with the exception that RMSEA and SRMR were slightly above the conventionally accepted benchmark. All item loadings were positive and significant at *p* < .001. In the African American subsample, latent variable loadings were 1.21 for PJW-others and .82 for DJW-others, and also 1.14 for PJW-self and .79 for DJW-self. In the White American subsample, latent variable loadings were 1.01 for PJW-self and 1.16 for DJW-self, and also 1.13 for PJW-others and .85 for DJW-others. For PA, even and odd parcels were .71 and .60 for African Americans, and .74 and .60 for White Americans. For NA, even and odd parcels were .71 and .59 for African Americans, and .55 and 1.03 for White Americans. The latent variable correlation between justice beliefs for self and others was .81 for African Americans and .73 for White Americans, while the PA and NA latent variable correlation was -.34 for African Americans and -.26 for White Americans.

**Table 4:** Justice beliefs for self and others predicting positive and negative affectivity: Multigroup model comparisons.

Model	χ^2^	*df*	NNFI	CFI	RMSEA	SRMR
1. Measurement Model	79.40	36	.94	.96	.09	.10
2. Fully Free Structural Model	42.20	28	.97	.99	.06	.05
3. Invariant Justice and Affectivity latent variable correlations	43.91	30	.98	.99	.06	.06
4. Invariant Justice for Others to Positive Affectivity	44.79	31	.98	.99	.05	.06
5. Invariant Justice for Self to Positive Affectivity	44.80	32	.98	.99	.05	.06
6. Invariant Justice for Self to Negative Affectivity	45.48	33	.98	.99	.05	.06
7. Invariant Justice for Others to Negative Affectivity	51.02	34	.98	.99	.06	.09
Model Comparisons	Δχ^2^		Δ *df*		*p*	
1 vs. 2	(37.20)		(8)		< .001	
2 vs. 3	1.71		2		*ns*	
3 vs. 4	.88		1		*ns*	
4 vs. 5	.01		1		*ns*	
5 vs. 6	.68		1		*ns*	
6 vs. 7	5.54		1		< .05	

Notes. NNFI = Non-Normed Fit Index; CFI = comparative fit index; RMSEA = root-mean-square error of approximation; SRMR = standardized root mean square residual. Value in parentheses indicates a decrease in Δχ^2^ and Δ *df*.

A fully free structural model (model 2) in which the four possible pathways from justice beliefs to affectivity were added and no invariance constraints were applied significantly improved fit of the initial measurement only model. Specifying equivalent latent variable correlations between self and other justice beliefs and also between PA and NA did not reduce model fit (model 3). Similarly, constraining the links from justice beliefs for self and others to PA did not reduce model fit (models 4 and 5). Although constraining links between justice beliefs for the self and NA did not reduce model fit (model 6), specifying equivalency of links between justice beliefs for others and NA did (model 7). We therefore settled on a final model in which self justice beliefs were equivalently linked to both PA and NA, and in which beliefs about justice for others were equivalently linked to PA but uniquely linked to NA (model 6).

**Fig 1:**
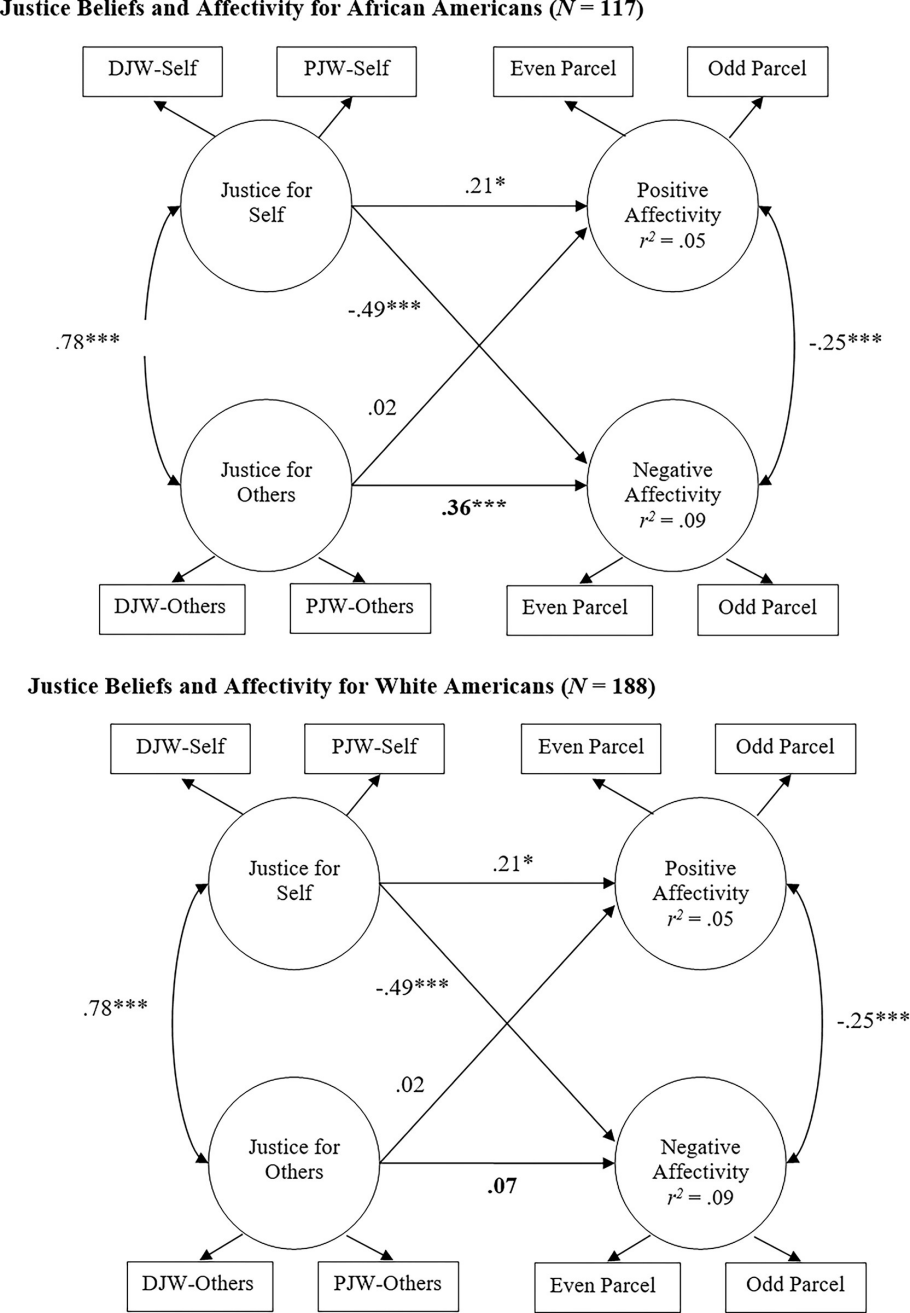
Path estimates for final structural model. Measurement model estimates excluded for ease of presentation. Estimate in bold indicates a non-invariant pathway. *p < .05, ***p < .001.

Fig 1 presents standardized estimates for the final model. As expected, and consistent with prior research, justice beliefs for self were associated with greater PA and reduced NA in both racial groups. Moreover, beliefs about justice for others were not associated with PA in either group. However, the hypothesized racial divergence was revealed by the links between beliefs about justice for others and NA. While there was no association with NA for White Americans, beliefs about justice for others were associated with greater NA among African Americans.

### Structural estimates and socioeconomic status

To assess whether ethnic differences in the link between justice beliefs for others and NA were explained by other social indicators, we conducted one final analysis in which we controlled for education and household income—our two markers of possible socioeconomic confounders. The final structural model (model 6) was reanalyzed using a partial correlations matrix, where variance due to education and income was removed [65]. The fit of this controlled model also was excellent, c^2^ (*df* = 33) = 45.47, *p* = .07, NNFI = .98, CFI = .99, RMSEA = .05 (90% CI: .00, .08), SRMR = .05. The salutogenic and invariant links between justice beliefs for self remained for both PA (.22, *p* < .05) and NA (-.47, *p* < .001). Moreover, there was once again no association between justice for others and PA (.02). Finally, while there was no association with NA for White Americans (.06), beliefs about justice for others were again positively associated with NA among African Americans (.47, *p* < .001).

## Discussion

Although believing in justice may confer benefits to personal well-being, recent theoretical and empirical work suggest that these relationships may be complex among racial minorities [9,11,28]. Guided by this emerging literature, we examined the extent to which beliefs about justice for self and others would be similarly and differently linked to positive and negative emotion among African Americans and White Americans. We expected that the belief in justice for self would be universally associated with better affectivity among both racial groups, but that beliefs about justice for others would be associated among African Americans only. Our findings were consistent with these predictions. Specifically, we found that the belief in justice for self was associated with greater positive affectivity and also reduced negative affectivity in both racial groups, whereas believing in justice for others was associated with higher negative affect only among African Americans.

To date, theory and research have largely emphasized the health enhancing effects of strong justice beliefs [37]. Along these lines, tendencies to believe in justice are seen as “healthy” through their capacity to promote better physical health and mental well-being, especially to the extent that justice beliefs can promote better stress coping. Our findings support this literature in the sense that believing one is personally fairly treated was associated with emotional well-being among African Americans and Whites alike, including through enhancing positive affect and reducing negative affect. Thus, our findings corroborate the notion that believing in personal justice may be generally health enhancing [66].

Importantly however, our findings also resonate with more recent empirical evaluations that have demonstrated beliefs about justice for others can also impact well-being among African Americans. Specifically, beliefs about justice for others may affect health behavior and stress coping among African Americans through either interactive or cooperative associations with personal justice beliefs [9]. The present findings align with this research to the extent that less strongly endorsing a belief in justice for others was associated with better emotional well-being for African Americans only. However, the present findings are distinguished to the extent that we found a robust main effect linking greater endorsement of the belief in justice for others to increased negative affectivity among African Americans. Thus, the present findings provide a unique contribution to this emerging literature in demonstrating an independent effect of beliefs about justice for others on emotional well-being, which did not depend on interactive associations with personal justice beliefs [9,43].

With respect to recent justice theory, the present findings resonate with inconsistency frameworks, which hold that individual well-being is governed by the extent to which one’s social expectations are concordant with one’s actual social experiences [11,42]. That is, agreement between one’s justice beliefs and justice-related experiences, rather than a rote endorsement of justice, fundamentally governs emotional health. Among racial minorities, who may be more often socially marginalized than majority group members, it follows that not endorsing some justice beliefs may be necessary to maximize consistency with lived experiences that do not as often reflect fairness. Thus, the present findings align with the seemingly ironic but key notion proffered by an inconsistency framework approach, which holds that less strongly endorsing a general belief in justice may better promote well-being in an unjust context [28].

The present findings may also be viewed in light of system justification theory, which holds that individuals seek to defend and justify the status quo–including social systems and structures that may disadvantage them–for the sake of psychological well-being [67]. Specifically, our findings suggest that system justification processes may be more connected to personal than general justice beliefs, and that justification may be balanced with rejection of some system justifying beliefs among racial minorities, such as African Americans. Finally, the present findings may be viewed in light of emerging research on primal worlds beliefs [68]. Specifically, whereas emerging primals research has suggested that demographic and socioeconomic variables account for little variability in positive world beliefs [69], the present findings suggest that such beliefs may nonetheless hold different functions for maintaining and protecting emotional well-being based on sociodemographic characteristics, such as race. Relatedly, we also found that justice beliefs were negatively associated with measures of household income and educational attainment, but only among African Americans.

With an eye towards practical applications, our findings may provide some useful insights for considering potential psychosocial interventions to better protect well-being among racial minorities. Such possibilities are buoyed to the extent that justice cognitions can be deliberately, readily, and precisely activated in individuals, [70,71]. Crucially, our findings suggest that crafting effective justice interventions for use with racial minorities might require intervention levers for attending to injustice-related thoughts and cognitions, as well as those related to justice [72]. However, we encourage caution in deploying health interventions that strive to alter justice cognitions, especially to the extent that imprecisely altering justice cognitions may produce ironic, unintended, and potentially harmful consequences [17].

Four general limitations suggest a cautious interpretation of results. First, we note that measurement model fit was moderate for RMSEA. To some extent, RMSEA values may reflect a relatively small sample size, coupled with the very large degrees of freedom that accompany a latent level analysis of a large measurement model. We are reassured to some extent by other fit indices, which were closer to adequate fit benchmarks and less impacted by large degrees of freedom. We further note that evaluation of these other fit indices (notably CFI) is recommended in considering measurement invariance. Second, the present study is correlational in nature. This limitation is offset by a large body of experimental and longitudinal research that has shown beliefs about justice act as a causal determinant of individual physical and emotional well-being [13,14,19,29,30]. Nevertheless, it is possible that these relationships are recursive, or that third variables not measured presently (e.g., perceived discrimination) also may contribute. Future experimental studies that specifically examine positive and negative emotion, and that measure additional third variables, may add further support to the directional associations modeled presently. A third limitation concerns including only a single (African American) minority sample. As such, it is not known whether the unique link between beliefs about justice for others and negative affectivity comprises a cultural phenomenon that is specific to African Americans, or whether this link may be generally observed among racial and other minority groups. Accordingly, future research also may examine whether links between beliefs about justice for others and emotional well-being comprise an emic versus etic phenomenon among minorities [46,73]. Finally, the present study did not assess cross-racial similarities and differences in links to social attitudes, which are also strongly connected to beliefs about justice. This includes evaluating potential divergent associations with beliefs about justice for self and others, with the latter typically providing more robust connections to social attitude measures in general participant samples. Future research should also consider social attitude measures, including social attitudes that could affect the well-being of self and others, such as interpersonal forgiveness [70].

## Conclusion

The present findings align with an emerging body of evidence by revealing that beliefs about justice hold a potential to both protect and detract from the emotional well-being of racial minority individuals. Specifically, the belief in justice for others was not associated with affectivity among White Americans, but was associated with greater negative affectivity among African Americans. The double-edged effect of believing in justice thus arises when beliefs about justice for oneself are parsed from a belief that the world in general is fair, as well as when positive and negative affectivity are separately and simultaneously considered. Double-edged connections to emotional well-being may be evident among racial minority groups such as African Americans because of past and ongoing experiences with social justice that have reinforced culture-specific associations linking thoughts about justice and injustice with emotional well-being. Better understanding how justice-related beliefs connect to mental and physical health among African Americans and other underserved minority groups and individuals may be critical to advancing psychological theory, and to developing justice-oriented social policies to address racial disparities.

## Supporting information

S1 File (CSV)

S2 File (CSV)
